# Relationship between the colours of the rivers in the Amazon and the incidence of malaria

**DOI:** 10.1186/s12936-023-04789-8

**Published:** 2023-11-23

**Authors:** Fernanda Fonseca, Jean-Michel Martinez, Antônio Balieiro, Jesem Orellana, James D. Santos, Naziano Filizola

**Affiliations:** 1 Instituto Leônidas e Maria Deane, Fiocruz Amazônia, Manaus, Amazonas Brasil; 2 Pós-Graduação em Clima e Ambiente—CLIAMB (INPA/UEA), Manaus, Amazonas Brasil; 3 Institut de Recherche pour le Développement, Toulouse, France; 4 Universidade Federal do Amazonas, Manaus, Amazonas Brasil

**Keywords:** Malaria, River colours, White water, Black water, Amazon

## Abstract

**Background:**

Malaria is transmitted by different *Anopheles* species. In Brazil, the disease is concentrated in the Amazon region. Rivers play an important role in the life cycle of malaria since the vector reproduces in aquatic environments. The waters of the rivers in the Amazon have distinct chemical characteristics, which affect the colour of the water and therefore, the study analysed whether the colour of the waters of the rivers have an on influence the distribution of malaria. The goal of the study was to correlate the different colourations of the water (black, white and mixed water) and the malaria incidence in 50 municipalities of the Amazonas state, Brazil, and then test hypotheses about the characteristics of the colour of the rivers and disease incidence.

**Methods:**

This study was conducted for a period of seventeen years (2003–2019) in 50 municipalities in the state of Amazonas, Brazil. A conditionally Gaussian dynamic linear model was developed to analyse the association of malaria incidence and three types of river colour: white, black and mixed.

**Results:**

The analyses indicate that the distribution of malaria is related to the colouration of the rivers. The results showed that places located near black-water rivers have a higher malaria incidence when compared to places on the banks of white-water rivers.

**Conclusions:**

Historically, the hydrological regime has played an important role in the dynamics of malaria in the Amazon, but little is known about the relationship between river colours and the incidence of the disease. This research was carried out in a region with hydrographic characteristics that were heterogeneous enough to allow an analysis that contrasted different colours of the rivers and covered almost the whole of the state of Amazonas. The results help to identify the places with the highest risk of malaria transmission and it is believed that they will be able to contribute to more precise planning of actions aimed at controlling the disease in the region.

## Background

Malaria is a disease that has a great impact on morbidity in tropical and subtropical countries and, in 2020, 241 million cases were registered worldwide [1]. In Brazil, the geographic distribution of the disease is heterogeneous, with variations over time and space, and areas with a high incidence rate and regions that are free of malaria or that have a low risk of transmission can be found [2, 3]. Most of the cases registered in the country are concentrated in the northern region and Amazonas is one of states with the highest incidence of the disease.

Though malaria is transmitted by different *Anopheles* species, the main vector of malaria in Brazil is *Anopheles (Nyssorhynchus) darlingi*. The mosquito normally reproduces on the margins of water bodies and adequate larval conditions depend on depth of the water, water flow, temporality (via rainfall seasonality), temperature, pH, chemical stability and light/shade proportions [4–7]. However, in high-density situations, the mosquito occupies several other types of breeding sites, such as small accumulations of water [4–6].

A variety of factors can contribute to the occurrence of malaria outbreaks and epidemics, including ecological conditions, poor sanitation, climatic conditions, environmental degradation and hydrological conditions [8–16]. Rivers play an important role in the life cycle of malaria, since the vector reproduces in aquatic environments [4–6]. The waters of the rivers of the Amazon have different chemical characteristics from other regions of the country, which is particularly due to the geology of the region, the type of vegetation, the presence of decomposing organisms and the climate [17–19].

The presence of suspended sediments in the rivers, which are generally sand, clay particles and silt, modify the characteristics of the water such as its physical–chemical properties and, in particular, the colour of the waters. These may either harm or favour the reproduction sites of malaria vectors and, consequently, have an impact on disease transmission.

In the Amazon, river colours are classified as either white, black or clear waters [17]. The white-water rivers were so named due to their origins in the Andes Mountains. They transport a large amount of suspended sediments and have a pH that is close to neutral. Among them, the Madeira, Purus, Juruá and Solimões/Amazonas Rivers stand out. Black-water rivers, on the other hand, get this name because of their characteristic colour, which results from the substances dissolved in them. Humic and fulvic substances stand out above all. These rivers have an acidic pH, carry a lot of organic matter and have a low concentration of suspended sediments in their waters. The largest of these is the Rio Negro, which originates between the Orinoco and Amazon River basins. Finally, the clear-water rivers (e.g., the Tapajós River) have either greenish colouration or are transparent. These rivers carry a small amount of dissolved sediments, have a certain level of dissolved organic matter and a pH that is close to neutral.

In this context, this study proposes to carry out an approach based on hypotheses, which correlate the colours of water in rivers in the Amazon and malaria incidence, in order to determine whether the characteristics of the type of water influence the distribution of the disease.

## Methods

Located in the northern region of Brazil, Amazonas is the largest state in the country and has an area of 1,559,167.878 km^2^. Although, in 2021, it had an estimated population of 4,269,995 million inhabitants that are distributed in 62 municipalities, the state of Amazonas has one of the lowest indices of demographic density in the country (2.23 inhabitants/km^2^, in 2010) [20].

**Fig. 1:**
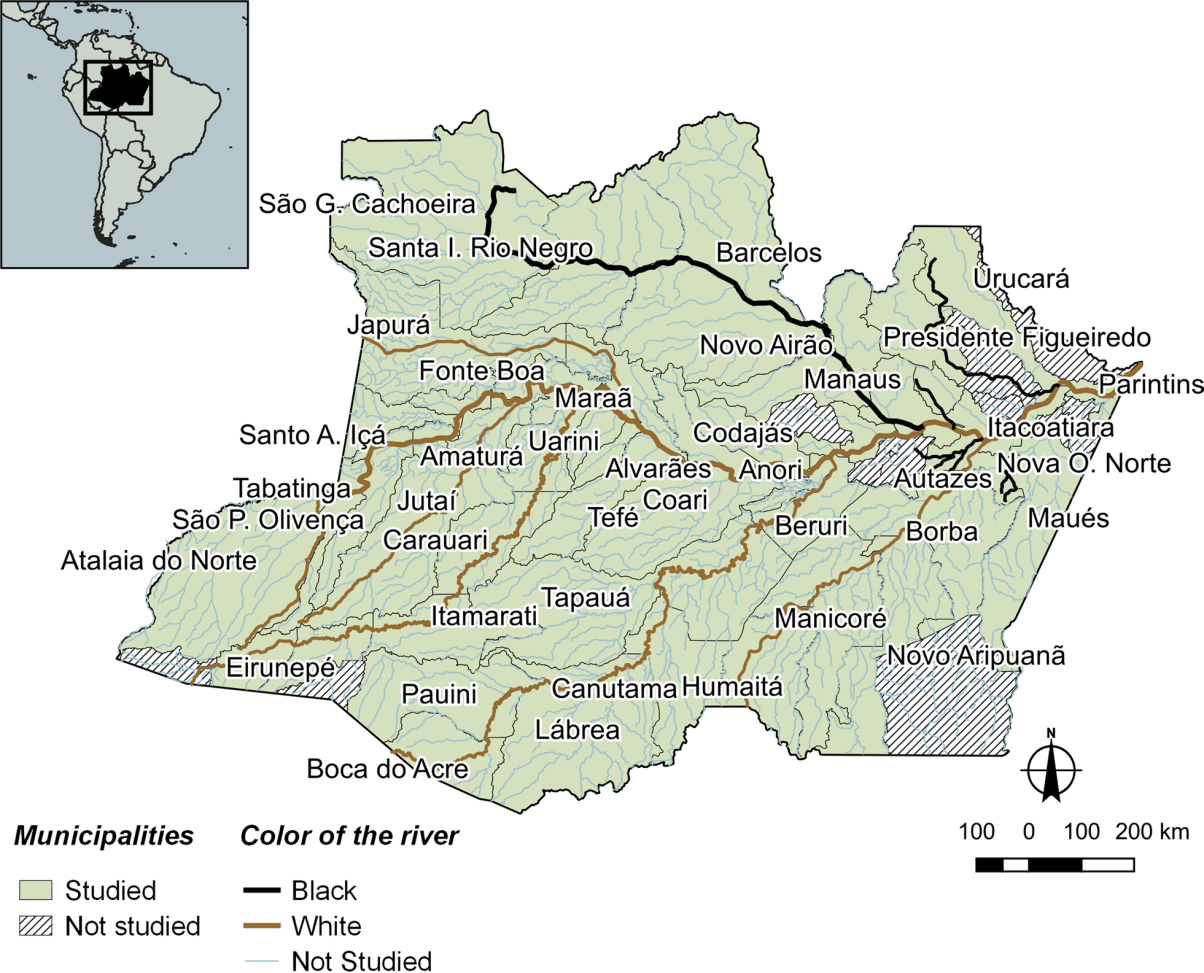
Area of study—Amazonas state, Brazil

The studied region has an extensive network of rivers and is within the largest hydrographic basin in the world, the Amazon basin. Of the 62 municipalities of the state of Amazonas, 50 municipalities were included in this study (Fig. 1) due to the availability of data for the variables studied, for being municipalities located on the banks of white-water or black-water rivers. Municipalities near clear-water rivers were excluded, due to the low number of them in the state of Amazonas.

In addition to rivers with waters of white or black colouration, in certain stretches of the rivers, there is the mixture of both colours. These watercourses were classified here as being of mixed waters. Due to the difficulty in spatially representing the beginning and end of mixed waters, this type of water class was not depicted in Fig. 1; however, they were considered in the characterization of the type of water colourations for each municipality investigated.

For the identification of the colour of the rivers, in addition to the visual interpretation carried out via satellite images using the Google Earth application, information from fluviometric stations belonging to the national hydrometeorological network (www.snirh.gov.br/hidroweb) and the database of the Observatory for Environmental Research on the Hydrology and Geodynamics of the Amazon Basin, ORE/HYBAM (www.ore-hybam.org) were also used. The classification of the colour of the waters of the rivers was based on the river where the fluviometric station closest to the headquarters of each municipality is found, in other words, the river closest to areas of the greatest human occupation.

Data on reported cases of malaria were obtained from the SIVEP-Malaria system, which is available to authorized users at http://www.saude.gov.br/sivep_malaria, after permission was granted to the authors of this research. Due to having digital records of the disease only from the year 2003 onwards, the historical series used in the study corresponds to the notifications of cases of malaria caused by *Plasmodium vivax* or *Plasmodium falciparum*, according to (most-probable) municipality of infection, between the years 2003 and 2019.

For comparison of malaria data between municipalities, and in order to evaluate the influence of the different types of colouration of the rivers in the Amazon, the data were standardized by calculating the annual parasitic incidence (API), as detailed in [20], whose formula is as follows:

1 $$API= \frac{{M}_{year}}{P} \times 1000$$

In which ${M}_{year}$ corresponds to the number of positive malaria tests, excluding the cure check testing, in the year, by probable municipality of infection, and *P* refers to the total resident population in the year considered. Estimated population data, from 2003 to 2019, were obtained from the Brazilian Institute of Geography and Statistics (IBGE, acronym in Portuguese). The cure check testing can be excluded, if necessary, since it represent cases of the same person. API is a commonly used indicator to analyse annual variations in positive laboratory tests for malaria in endemic areas, as part of the group of epidemiological surveillance actions for the disease.

For data analysis, the municipalities were divided according to the colour of the water of their respective rivers. Then, for each year and colour type, the log-API density function was estimated via the kernel method. Due to the high API variability, log-API was used in the analysis as it allowed the use of normal mixtures in modelling.

Let $k\in \left\{white,mixed,black\right\}$ be a variable that indicates the colour of the river. Let ${\mathcal{T}}_{k}$ be a set of indices for municipalities with river of colour $k$. Let ${y}_{i,t}^{\left(k\right)}$ be the value of log-API for the municipality $i$, with $i\in {\mathcal{T}}_{k}$, at the time $t$. Given the previous discussion, it is reasonable to assume that the log-API can be explained by the following mixture model

2 $$f\left({y}_{i,t}^{\left(k\right)}|{\mu }_{1,t}^{\left(k\right)},{\mu }_{2,t}^{\left(k\right)},{\sigma }_{1}^{\left(k\right)},{\sigma }_{2}^{\left(k\right)},{p}^{(k)}\right)={p}^{(k)}\phi \left({y}_{i,t}^{\left(k\right)}|{\mu }_{1,t},{\sigma }_{1}^{\left(k\right)}\right)+\left(1-{p}^{(k)}\right)\phi \left({y}_{i,t}^{\left(k\right)}|{\mu }_{2,t},{\sigma }_{2}^{\left(k\right)}\right),$$

Where $\phi \left(.|a,b\right)$ is the density function of the normal distribution with mean $a$ and standard deviation $b$. Every mixture model admits a representation from an augmented model, considering the latent variable ${z}_{i}^{(k)}\sim Bernoulli\left({p}^{(k)}\right)$, which implies that ${y}_{i,t}^{\left(k\right)}$ comes from the model $\pi \left(.|{\mu }_{1,t}^{(k)},{\sigma }_{1}^{\left(k\right)}\right)$ with probability ${p}^{(k)}$ or it comes from $\pi \left(.|{\mu }_{2,t}^{(k)},{\sigma }_{2}^{\left(k\right)}\right)$ with probability ${1-p}^{(k)}$. Conditioned to ${z}_{i}^{(k)}$, for all ${i\in \mathcal{T}}_{k}$, it is true that the observation model has normal distribution, i.e.,

3 $$f\left({y}_{i,t}^{k}|{z}_{i}^{\left(k\right)},{\mu }_{1,t}^{\left(k\right)},{\mu }_{2,t}^{\left(k\right)},{\sigma }_{1}^{\left(k\right)},{\sigma }_{2}^{\left(k\right)}\right)=\phi \left({y}_{i,t}^{\left(k\right)}|{z}_{i}^{(k)}{\mu }_{1,t}^{\left(k\right)}+\left(1-{z}_{i}^{(k)}\right){\mu }_{2,t}^{\left(k\right)},{\sigma }_{1}^{(k){z}_{i}^{(k)}}{\sigma }_{2}^{(k)\left(1-{z}_{i}^{(k)}\right)}\right)$$

Therefore, it can be assumed that ${\mu }_{j,t}^{\left(k\right)}|{\mu }_{j,t-1}^{\left(k\right)}\sim {\rm N}\left({\mu }_{j,t-1}^{\left(k\right)},{w}_{j}\right),$ for $j=\mathrm{1,2}$. Adding conditional independence between ${\mu }_{1,t}^{\left(k\right)}$ and ${\mu }_{2,t}^{\left(k\right)}$ to this last structure, the problem comes down to a conditionally Gaussian dynamic linear model (CGDLM) [21]. In this way, the posterior inferences for the levels can be carried out using a Gibbs sampler, whereby the complete conditional of the levels is obtained via forward filtering backward sampling [22]. The variances through the inverse gamma distribution and the probability of the mixture and latent variables can be obtained via the beta and Bernoulli distribution, respectively [23].

The analyses were performed in the R software (version 4.3) in the RStudio development environment (version 1.4.17). As a decision-making tool, the posterior probability used in all statistical tests was 0.95.

## Results

**Fig. 2:**
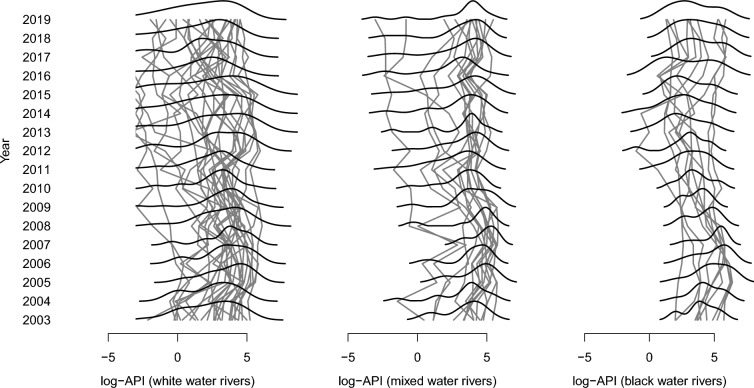
Time series of the log-API, for each municipality, considering the colour of their respective rivers

Figure 2 shows the time series of the log-API, for each municipality, considering the colour of their respective rivers. It can be noted that white-water rivers have greater variability in log-API values. Furthermore, there is a dominant and positive mode over time, close to value 4, representing most municipalities and another smaller one, with typically negative values and varying over time. The same behaviour can be noted for municipalities with mixed water. The municipalities with black water tend to have two modes with similar values, with log-API values between 3 and 5.

**Fig. 3:**
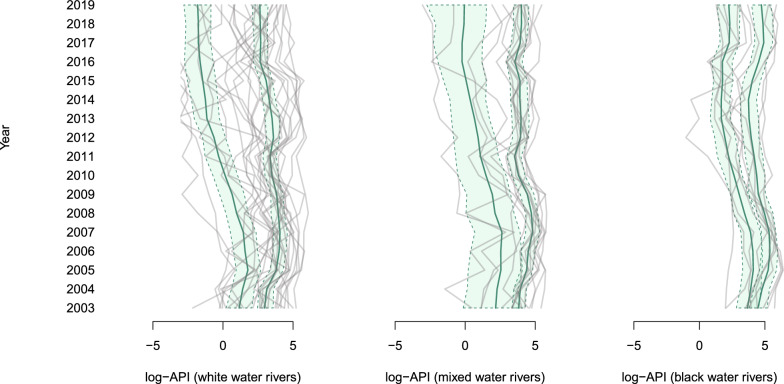
Gray lines: observed log-API values over time for each municipality. In blue: the polygons represent the 95% confidence interval for the log-API level for each detected class, while the solid line within the polygon represents the average level

Figure 3 shows the smoothed average of the log-API level, with the respective 95% credibility intervals, for the two mixture classes, considering the grouping of municipalities by colour of the water. For the three groupings, it can be noted that the average levels distanced themselves over time.

**Fig. 4:**
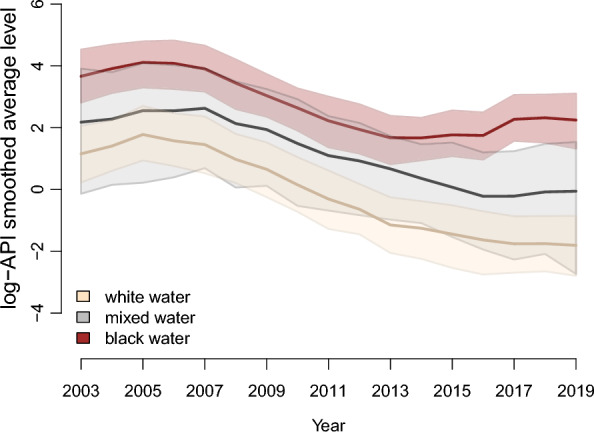
95% credibility intervals and estimates for the log-API level referring to the smallest class detected for each group of municipalities by colour of their respective rivers

The results shown in Fig. 4 indicate the log-API level, considering only the mixture class with the lowest value for each grouping. It is possible to note that the average levels for white- and mixed-water rivers are within each other's credibility interval. In fact, the posterior probability that the log-API level for the mixed-water class is greater than that of white water is only 40.15%, which is an insufficient value to allow us to accept the hypothesis of a difference between classes. The same does not occur with the average level of black-water rivers, which have a higher value than the other two, with the posterior probability that the log-API level for the black water is greater than the white water being 99.91% and that it is greater than the mixed-water class being 99.18%.

**Fig. 5:**
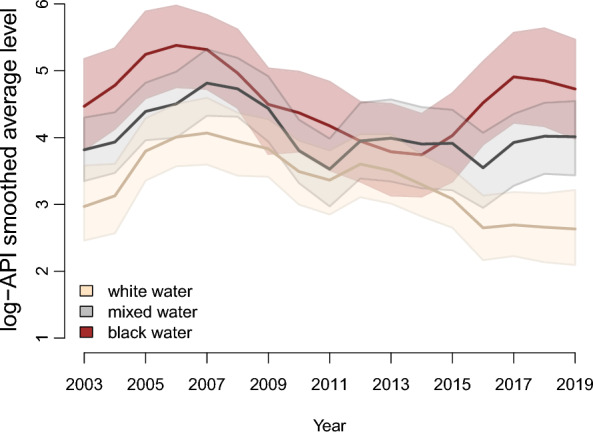
95% credibility intervals and estimates for the log-API level referring to the largest class detected for each group of municipalities by colour of their respective rivers

Figure 5 shows the log-API level, considering only the mixture class with the highest value for each grouping. Although the intervals overlap, there is a hierarchical order in the average, with black-water rivers having the highest values, followed by mixed- and white-water rivers.

The posterior probability that the average log-API level for mixed water is higher than that for white water being 88.75% (a high value, but not enough to accept the difference). A similar value is obtained by calculating the probability that the log-API level for mixed water is lower than that for black water (88.11%). The probability that the log-API level for white water is lower than black water is 96.45%, which leads us to accept this difference.

Finally, the posterior probability of the log-API level of the largest mixed class, considering the group of municipalities with white-water rivers being lower than the smallest black water mixed class, is 0.29. Therefore, there is no reason to believe that the levels in these classes are different.

The analyses suggest that, within the class with the lowest log-API level, 26% of white-water municipalities and 22% mixed-water municipalities have a decreasing trend. In the class with the highest log-API level, the values remain relatively constant for both river water colours. In black-water rivers, in 100% of the municipalities, the log-IPA remained relatively constant.

Therefore, the results indicate that the influence on the distribution of malaria is differentiated between the colouration of the rivers. The results show that municipalities that are located on the banks of black-water rivers have a greater malaria incidence when compared to those on the banks of white-water rivers.

## Discussion

The decrease in malaria over the years has also been reported in other studies in the Amazon and around the world [1, 24, 25]. In recent decades, government initiatives have driven the progress in combating malaria, and this has resulted in reductions in the incidence of the disease and, thus, reductions in severe cases and deaths. Since 2000, there has been a significant increase in the number of countries that have achieved the elimination of malaria [26]. As has occurred in the global scenario, the downward trend in cases of the disease in Brazil can be explained by the prioritization in the national health policy agenda regarding the need to prevent a sharp increase in the disease. In the state of Amazonas, in 2007, there was a large investment in malaria prevention, control and surveillance actions, which boosted the progress in reducing malaria cases in the state. At that time, the multi-annual plan of malaria control actions in the state of Amazonas (PPACM 2007/2010) was implemented, which was coordinated by the Health Surveillance Foundation (HSF). According to the HSF, the PPACM established the agreement between the municipalities of the state regarding the goal of achieving, by 2010, an 80% reduction in malaria compared to 2007. Great success was achieved during this period [24].

The hydrological cycle has shown a fundamental role in relation to the dynamics of malaria [14, 15]. The results reveal that the type of colouration of river waters is a relevant factor that contributes to the disease incidence. Other studies have also reinforced the influence of the colour of the rivers in the Amazon on the malaria incidence [27], indicating that, in addition to the oscillation in the level of the rivers, the difference in seasonality of the disease may be linked to the colour of the waters of the rivers, with a more marked seasonality in municipalities on the banks of white-water rivers. In municipalities on the banks of black-water rivers, seasonality was not very evident. The authors also point out that malaria is more intense in places and periods of the hydrological cycle with low concentrations of suspended sediments in rivers, this being one of the appropriate conditions for the development of the disease vector cycle.

The findings of this work also agree with Tadei et al*.* [28, 29]. These authors indicated that the Negro River, which has black, acidic waters, low productivity, low concentrations of suspended sediments and low electrical conductivity, provides a favourable environment for the development of *An. darlingi*. Suspended sediments in rivers are one of the factors that modify the physicochemical characteristics of waters, and cause a change in their colour, a decrease in water temperature and an increase in turbidity, pH and conductivity.

According to Laraque et al*.* [30], the Negro River has low values for suspended sediment concentration throughout the year. These concentrations represent less than 0.1% of those found in the Solimões River. In addition, the Negro River presents values of other parameters that are also lower when compared to the Solimões River, such as speed (0.3 vs. 1 m/s), conductivity (8 vs. 80 µS/cm at 25 °C), turbidity (5 vs. 80 NTU) and pH (5.5 vs. 7.0); and higher values for temperature (1 °C) [30]. Thus, it is suggested that the characteristics of black waters when compared to white waters may provide even more suitable conditions for the presence of the malaria vector, which influences the disease incidence in places on the banks of these rivers.

Although the trends indicated in the results of this study and the method and analysis indicated a good relationship with other studies discussed above, it is necessary to highlight some limitations. The use of secondary malaria notification records, which are subject to underreporting and often have poor quality of records, are some of the limitations that deserve attention. Secondly, the analysis was not adjusted for variables that may be distorting the interpretation of the results. In addition, the categorization strategy of the data referring to the variable colouration of the rivers was generalized due to the operational difficulty of classifying these waters in municipalities that have a significant geographical extension and hydrographic particularities that are typical of the largest hydrographic basin in the world.

Nonetheless, this study presents an unprecedented approach regarding the analysis of water colour and the malaria incidence. In addition, it was carried out in a region with hydrographic characteristics that were heterogeneous enough to allow an analysis that contrasted different colours of the rivers and covered almost the entire state, which has the highest malaria incidence in Brazil. Thus, it is believed that these results may contribute to the more precise planning of actions aimed at disease control.

## Conclusions

The study sought to analyse whether the colour of the waters of the rivers in the Amazon influence the distribution of malaria. The results showed a decreasing trend of the disease over the years from 2003 to 2019, in all types of rivers (white, black and mixed water). Furthermore, the dynamic model (CGDLM) generated the following evidence: (1) any class in the black-water group has a higher mean log-API level compared to other classes of other colours; (2) there is no difference between the smallest classes of white- and mixed-water rivers; (3) there is no difference between the class with the average level from the mixed-water group and the other classes with higher averages; (4) the largest black-water class has higher levels than the white-water class.

## Data Availability

Malaria data in SIVEP-Malaria (http://www.saude.gov.br/sivep_malaria). River data: National Hydrometeorological Network (www.snirh.gov.br/hidroweb) and the database of the Observatory for Environmental Research on the Hydrology and Geodynamics of the Amazon Basin, ORE/HYBAM (www.ore-hybam.org).
